# Physics of microstructures enhancement of thin film evaporation heat transfer in microchannels flow boiling

**DOI:** 10.1038/srep44745

**Published:** 2017-03-17

**Authors:** Sajjad Bigham, Abdolreza Fazeli, Saeed Moghaddam

**Affiliations:** 1 Department of Mechanical and Aerospace Engineering, University of Florida, Gainesville, FL 32611, USA

## Abstract

Performance enhancement of the two-phase flow boiling heat transfer process in microchannels through implementation of surface micro- and nanostructures has gained substantial interest in recent years. However, the reported results range widely from a decline to improvements in performance depending on the test conditions and fluid properties, without a consensus on the physical mechanisms responsible for the observed behavior. This gap in knowledge stems from a lack of understanding of the physics of surface structures interactions with microscale heat and mass transfer events involved in the microchannel flow boiling process. Here, using a novel measurement technique, the heat and mass transfer process is analyzed within surface structures with unprecedented detail. The local heat flux and dryout time scale are measured as the liquid wicks through surface structures and evaporates. The physics governing heat transfer enhancement on textured surfaces is explained by a deterministic model that involves three key parameters: the drying time scale of the liquid film wicking into the surface structures (τ_d_), the heating length scale of the liquid film (δ_H_) and the area fraction of the evaporating liquid film (A_r_). It is shown that the model accurately predicts the optimum spacing between surface structures (i.e. pillars fabricated on the microchannel wall) in boiling of two fluids FC-72 and water with fundamentally different wicking characteristics.

Flow boiling process is a ubiquitous thermal transport technique that has played a key role in many technological and industrial applications for more than half a century. The unique ability of this mechanism in transferring substantial amount of heat from a heated surface has enabled critical technologies ranging from HVAC systems to large boilers that power electricity generation plants. In recent years, major advancements of manufacturing technologies in areas such as power and RF electronics, high performance computers and solid-state lasers have led to extremely high heat generation rates in small volumes^1^,^2^,^3^,^4^ which in turn have prompted the scientific community to implement the flow boiling process in microchannels^5^,^6^,^7^,^8^,^9^,^10^,^11^. To achieve this goal, numerous studies have been conducted to characterize and enhance heat transfer performance of microchannel heat sinks. These studies^4^,^12^,^13^,^14^,^15^,^16^,^17^,^18^,^19^ have identified different mechanisms responsible for heat transfer enhancement in the microchannel flow boiling process. Liquid rewetting of the surface^4^,^14^,^15^,^18^, bubble nucleation and growth^4^,^14^,^15^,^16^, and thin film evaporation^12^,^13^,^17^ can have a significant impact on heat transfer in microchannels, depending on the flow regime, wall temperature and input heat flux.

Integration of surface structures on the microchannels walls has been suggested as a means of enhancing the heat transfer coefficient and the critical heat flux (CHF). These structures have been proven to enhance the heat transfer coefficient and the CHF in the pool boiling process^6^,^7^,^8^,^9^,^10^,^11^,^20^,^21^,^22^,^23^. The reported enhancement in heat transfer and CHF was mainly attributed to improved surface wickability^10^,^21^, increase in active nucleation sites^23^ and enhanced heat transfer area^8^,^9^,^22^ associated with such structures. However, their implementation on microchannels walls has produced mixed results ranging widely from a decline to a substantial increase in performance. Li *et al*.^6^ were among the first to report utilization of silicon nanowires on microchannels walls. They fabricated a heat sink with microchannels that were 250-μm-wide, 200-μm-deep, and 20-mm-long and conducted tests with water at a mass flux range of 119 to 571 kg/m^2^.s. The nanowires primarily impacted the heat transfer coefficient without a significant improvement in the maximum surface heat flux (reported to be 115 W/cm^2^ at a mass flux of 571 kg/m^2^.s) compared to that of a plain surface. The implementation of nanowires enhanced the heat transfer coefficient at mass fluxes of 238, 357 and 571 kg/m^2^.s; however, at the lowest mass flux of 119 kg/m^2^.s, the heat transfer coefficient was inferior to that of the plain-surface microchannels. The authors suggested that substantial ebullition from the nanowires at the microchannel entrance resulted in a rapid transition of flow to an annular flow regime. The authors then argued that at the higher mass fluxes, a liquid film forms more easily on the microchannel surface and is able to persist over the duration of the annular flow resulting in enhanced boiling heat transfer. But, at the lowest mass flux, the liquid film disappears more quickly, and the consequent dryout leads to an inferior heat transfer performance to that of the microchannels with plain walls.

Silicon nanowires were similarly grown in 200-μm-wide, 250-μm-deep, and 10-mm-long microchannels in a subsequent study by Yang *et al*.^24^. Tests were conducted at a mass flux range of 113 to 389 kg/m^2^.s. Yang *et al*.^24^ also suggested that substantial generation of bubbles at the channel entrance resulted in a quick transition to an annular flow regime but unlike Li *et al*.^6^ they observed significant enhancement in CHF by using hydrophilic silicon nanowires. Unfortunately, Yang *et al*.^24^ did not report the surface temperature and heat transfer coefficient at different operating conditions hence a careful comparison with findings of Li *et al*.^6^ cannot be conducted. Yang *et al*.^24^ high-speed imaging studies showed that the suggested annular flow regime has a periodic nature (due to a highly sub-cooled inlet conditions) with a frequency on the order of 20 ms. They argued that the vapor core formed within the channel rapidly grows and eventually approaches the microchannel walls. As a result, the unsaturated nanowires in the downstream instantly act as wicking structures to activate capillary flows along the channels walls. In a more recent study, through an analysis of different forces (inertia, surface tension, shear, buoyancy and evaporation momentum forces), Alam *et al*.^25^ argued that the enhanced surface rewetting and CHF in microchannels with nanowires are owing to a higher surface tension force at the liquid–vapor interface. They argued that a low contact angle on walls with nanowires results in a higher surface tension and therefore a lower Weber number compared to the flow in a microchannel with a plain surface, leading to a uniform and more stable liquid film over a surface with nanowires.

Nanowires have also been utilized to enhance the flow boiling heat transfer performance of the FC-72 liquid. Shin *et al*.^26^ studied the effect of nanowires with a height ranging from 0.8 μm to 15.7 μm at different surface superheat temperatures and heat fluxes. The results indicated a decline in CHF in all test conditions. The changes in the heat transfer coefficient followed a complicated pattern ranging from a decline to enhancement depending on the nanowires height, flow Re number, and surface superheat temperature and heat flux. The authors argued that enhanced nucleation as well as convective effects could be responsible for the observed enhancement of the surface heat transfer coefficient in the case of long nanowires.

Evidently, the existing literature is lacking a mechanistic description of the role of surface structures on the microchannel flow boiling heat transfer process. Here, through experiment and modeling, it is clearly shown how surface structures impact a key mechanism of heat transfer in the flow boiling process (i.e. thin film evaporation) and provide a guideline for designing the surface structures. In the following section, first, a unique experimental approach is employed to identify details of the individual heat transfer events in the microchannel flow boiling heat transfer process. The evaporation time scale of the liquid film formed within the surface structures is directly measured in an experiment involving a single instrumented microchannel and controlled flow conditions. A model is then introduced that provides the key parameters dictating the flow and evaporation of the liquid film within the surface structures. Finally, a second experimental study is introduced to demonstrate the efficacy of the proposed model in predicting the optimal spacing of the patterned surface structures in a typical microchannel heat sink.

## Experimental characterization of thin film evaporation process

Recently^12^,^13^, the authors have studied the thermal field underneath a moving bubble and demonstrated that the evaporation of a thin liquid film formed on the microchannel surface is a key heat transfer mechanism. This process is hypothesized to be affected when the surface of a microchannel is patterned. Hence, an experiment is designed to directly measure the local surface heat flux and the dryout time of the thin liquid films formed on a plain as well as five textured surfaces.

### Experimental procedure

**Figure 1:**
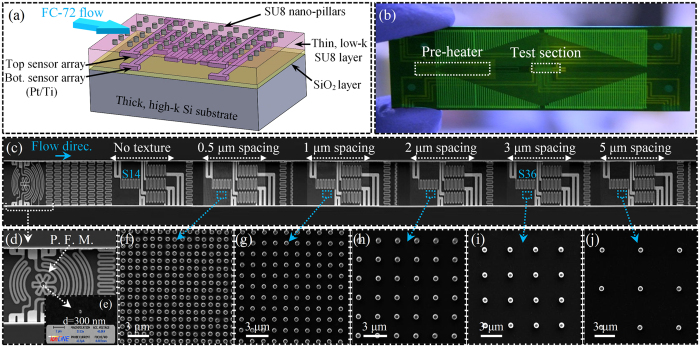
(**a**) A cross-sectional view of the composite substrate and SU8 pillars; (**b**) an image of the microfluidic chip (pre-heater and test sections are labeled); (**c**) an SEM image of the test section; (**d**) an SEM image of pulsed function microheater; (**e**) an SEM image of a 300 nm cavity fabricated by FIB at the center of the pulsed function microheater, and (**f–j**) top-view SEM images of pillar structures with different pillars spacings.

A unique experimental approach is utilized which is capable of measuring the local heat flux. Details of the test article are shown in Fig. 1. The test device consists of two temperature sensor arrays (top and bottom RTD arrays depicted in Fig. 1a), separated by a thin layer of a low thermal conductivity material (a 10-μm-thick SU8 polymer layer), sputter deposited on a thick high thermal conductivity silicon substrate. This sensor arrangement, explained comprehensively in authors’ previous studies^12^,^13^,^27^,^28^,^29^,^30^, is used to determine the local heat flux with a spatial resolution of 20–50 μm and a temporal resolution of 50 μs. These sensors are calibrated prior to the experiments in a uniform temperature oven to obtain the resistance-temperature relationship of each sensor. Considering the sensitivity of the sensors and the data acquisition system (DAQ) uncertainty, the maximum error in temperature measurements is determined to be ±0.25 °C.

The test platform is a microfluidic chip (Fig. 1b) with a single microchannel at a height of 75 μm and a width of 250 μm. The channel is capped by an optically transparent Polydimethylsiloxane (PDMS) layer. The surface structures (i.e. pillars) are fabricated on top of the sensor layers, as depicted in Fig. 1a. The structures are made from SU8 through spin coating and using the electron-beam lithography (EBL) technique. SEM images of the pillars are provided in Fig. 1c and f–j. A total of five structures with pillar spacings 0.5, 1, 2, 3, 5 μm and a diameter of 500 nm and a height of ~2 μm are fabricated along the flow direction. The tests are conducted with FC-72 liquid at saturation conditions.

In the experiments, temperature data and synchronized bubble images are recorded at a frequency of 20 kHz. To control the nucleation site, a 300 nm in diameter cavity is fabricated using a focused ion beam (FIB) milling machine. The cavity is surrounded by a pulsed function microheater (cf. Fig. 1d,e) that is physically connected to a programmable dc power supply module. This configuration allows us to generate bubbles at a desired frequency and size. The thin film heaters are also powered by the NI dc power supply. All data collection, as well as the control for the applied dc voltages of the heaters, are performed using a LabVIEW program.

A high-speed camera (FASTCAM SA4-Photron) is synchronized with the DAQ to visualize the boiling process at a frequency of 20 k frames per second. The working fluid is delivered to the microfluidic chip by a piezoelectric micropump (Model MP6, manufactured by Bartels Mikrotechnik GmbH). Two PX-26 pressure transducers with ± 1% reading error are used to measure the pressure drop across the microchannel. The working fluid is degassed by vigorous boiling for several hours before each experiment. Then, the desired surface temperature is adjusted and allowed about 15 minutes to reach a steady state before recording the data. Further details of the fabrication process, experimental procedures, calibration tests, and uncertainty analysis are presented in the Supplementary Materials section.

### Analysis of experimental data

**Figure 2:**
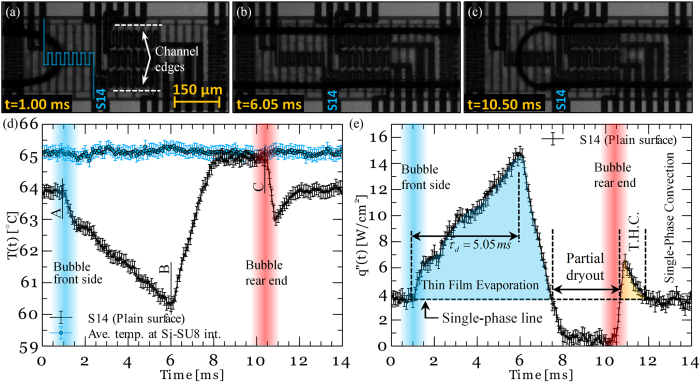
(**a–c**) Images of a bubble, pictures are taken using FASTCAM SA4-Photron high speed camera at a frequency of 20k frames per second with picture resolution of 512 × 256 pixels; (**d**) surface temperature-time history; (**e**) local surface heat flux-time history corresponding to a bubble flowing on a plain surface. Test is conducted at a mass flux of 73.8 kg/m^2^-s. T.H.C. stands for transient heat conduction.

To understand the role of surface patterns, the heat transfer events on a plain surface are first analyzed. This analysis is accomplished by evaluating the thermal field at sensor 14 located at the center of the channel where the local dryout is initially expected. Fig. 2a–c provide images of a moving bubble during its growth over the sensor. The corresponding local temperature and heat flux data are provided in Fig. 2d,e. The blue and red shadow lines indicate the times at which the front and rear ends of the bubble arrive at the sensor footprint, respectively. Comparison of the bubble images with the corresponding temperature and heat flux data shows that the local heat flux at the center of the microchannel spikes up to ~14 W/cm^2^ after the front side of the bubble arrives at the sensor footprint. This surface cooling effect lasts for more than 5 ms during which the bubble leading edge has long past over the sensor footprint. Therefore, the observed heat flux rise is due to the evaporation of a liquid layer formed on the surface as a result of the preceding liquid slug flow. After τ = 5.05 ms, the local heat flux reaches its maximum when the onset of dryout occurs. This time period is considered to be the drying time scale (τ_d_) of the surface.

As it can be seen, the surface experiences a second spike in heat flux as the rear end of the bubble rewets the sensor footprint. This sudden spike in the local heat flux resembles observations made in pool boiling studies during the bubble departure, as the liquid front advances over (i.e., rewets) the bubble-surface contact area after a dryout period following the microlayer evaporation process. The physics of this process is consistent with what is commonly named as the “transient conduction” mode of heat transfer, which results from the rewetting of a hot surface with the cooler bulk liquid. After the liquid slug fully rewets the surface, the local heat flux gradually decreases and approaches that of the single-phase heat transfer. As the results indicate, the nature of heat transfer is predominately governed by the thin film evaporation mode of heat transfer.

**Figure 3:**
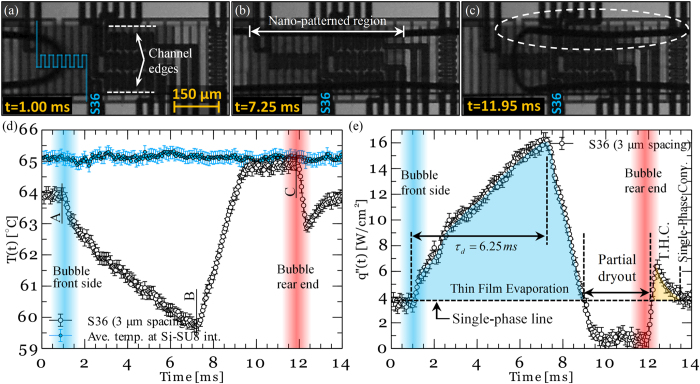
(**a–c**) Images of a bubble, pictures are taken using FASTCAM SA4-Photron high speed camera at a frequency of 20k frames per second with picture resolution of 512 × 256 pixels; (**d**) surface temperature-time history; (**e**) local surface heat flux-time history corresponding to a bubble flowing on a surface with a pillar edge-to-edge spacing of 3 μm. Test is conducted at a mass flux of 73.8 kg/m^2^-s. T.H.C. stands for transient heat conduction.

Figure 3a–c show images of the bubble shown in Fig. 2 as it flows over a surface with a pillar edge-to-edge spacing of 3 μm (shown in Fig. 3b). A comparison of the bubble images shown in Figs 2c and 3c indicates that the three-phase line is pulled further into the channel providing a visual verification of the effect of pillars on the liquid distribution. The corresponding local temperature and heat flux data measured at sensor 36 are shown in Fig. 3d,e. Sensor 36 as well as similar sensors underneath the individual structured regions provide a direct measurement of the drying time scale during the combined wicking-thin film evaporation process during which the liquid volume left at the sides of the microchannel is wicked over a length scale of ~125 μm (i.e. half of the microchannel width). While the heat transfer event observed on the patterned surface is very similar to that observed on a plain surface, the measured heat flux and drying time of the surfaces differ. A comparison of the drying time scales observed in Figs 2e and 3e suggests that a liquid film formed on a textured surface takes a longer time to evaporate compared to a liquid film formed on the plain surface. This observation shows that the capillary wicking effect induces the liquid micro-inflows within the pillars during the thin film evaporation process leading to a longer drying time scale. Also, the results clearly show that the thermal characteristics of the other heat transfer events (i.e. transient conduction and convective heat transfer to the liquid phase) are almost identical to those observed on the plain surface.

## Modeling of thin film evaporation heat transfer process

### Model development

In the previous section, it was shown that during the flow boiling process in a microchannel vapor slugs form and interrupt the otherwise continuous liquid flow stream. Here, it is assumed that the liquid and vapor slugs are flowing at a velocity of U to estimate the exposure time of the heat transfer surface to a bubble (i.e., τ^*^ ~ L/U) where L denotes the bubble length (see the Supplementary section for more details). At a bubble generation frequency of *f = 1/τ*, τ^*^/τ and 1-(τ^*^/τ) are time fractions associated with the surface exposure to vapor and liquid slugs, respectively. When the channel is occupied by very short vapor slugs generated at a low frequency, the numerical value of τ^*^/τ is negligible (i.e., the flow boiling heat transfer is dominated by heat transfer to the liquid slug, 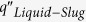). However, when long vapor slugs are generated at a high frequency, τ^*^/τ approaches unity (i.e., the flow boiling heat transfer is dominated by heat transfer mechanisms associated with the vapor slug, 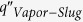). Using this analogy, the overall surface heat transfer (*q“*_*T.Ph.*_) can be written as follows:


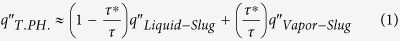


In the test conditions of this study, heat transfer associated with the liquid slug can be predicted fairly accurately using the single-phase laminar flow theory. This has been discussed in details in authors’ prior studies^12^,^13^. The thermal events associated with the vapor slug flow are the thin film evaporation and the surface partial dryout processes. Let us consider *τ*_*d*_ as the time scale of the liquid film evaporation (i.e., the drying time scale). Therefore, τ_d_/τ^*^ and 1-(τ_d_/τ^*^) represent time fraction of the heat transfer surface exposure to the thin film evaporation and the dryout events, respectively. Hence, the surface heat transfer associated with the vapor slug flow can be presented as:


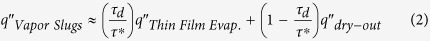


**Figure 4:**
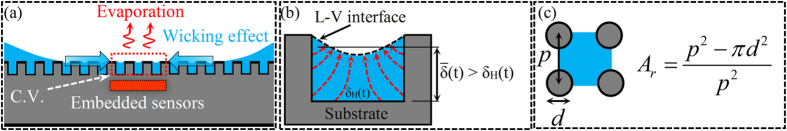
Schematics showing cross-sectional and top views of surface pillars depicting (**a**) liquid wicking and evaporation, (**b**) heat flow from the walls to the liquid-vapor interface and (**c**) difference between the overall wick projected and evaporation areas.

**Figure 5:**
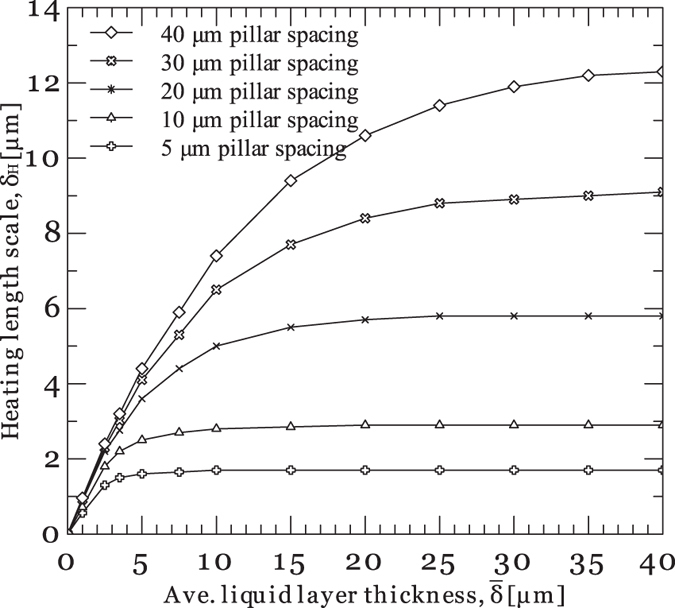
Heating length scale (*δ*_*H*_(*t*)) as a function of average liquid film thickness and pillars spacing.

Figure 4a,b depict the liquid film wicking and evaporation on a textured surface as well as the meniscus formed between surface micropillars, respectively. An average liquid layer thickness (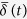) is assumed that continuously varies during the thin film evaporation process. The heating length scale, (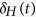), is also introduced which factors in the fact that extended surfaces shorten the heat transfer path to the liquid-vapor interface. To determine the heating length scale, a 3D conduction heat transfer equation is numerically solved. The heating length scale is then defined as 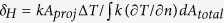 where 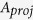 and 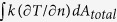 are the projected liquid-vapor interface area and the total heat transfer from the solid area to the liquid, respectively. The simulation results shown in Fig. 5 relate the heating length scale to the average liquid layer thickness and the pillar edge-to-edge spacing. The heat flux associated with the thin film evaporation process can then be estimated by 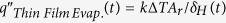 where *A*_*r*_ is the ratio of the projected liquid-vapor interface area to that of a unit cell between the pillars (cf. Fig. 4c). By combining Eqs (1) and (2) and neglecting the small heat transfer during the partial dry-out period, the two-phase heat transfer can be determined as:


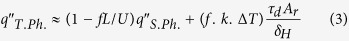


where *f* is the bubble generation frequency (i.e., *f* = 1*/τ*). The bubble generation frequency and parameters 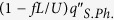 and *f.k.*Δ*T* are almost constant at a given test conditions. Therefore, the above equation suggests that the two-phase heat transfer has a linear relationship with parameter 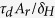 with a proportionality constant of *f.k.*Δ*T*. The drying time scale associated with the evaporation of the liquid film fed by the capillary induced micro-inflows can be determined by applying a mass balance to the control volume shown in Fig. 4a (i.e., *dM*_0_/*dt* = 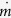
_*wick*_ −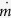
_*evap*_). Through this approach, an equation was developed which could express variations in the average liquid layer thickness during combined wicking-thin film evaporation process as follows:


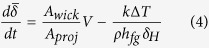


where *A*_*wick*_ and *A*_*proj*_ are the cross-sectional area available for wicking and the area over which the liquid is spread, respectively. *V* is the mean imbibition velocity of the liquid wicked into the surface structures. As mentioned earlier, capillary pressure induced by narrow gaps within the surface structures generates micro-inflows that replenishes the liquid vaporized during the thin film evaporation process. However, viscous losses associated with the flow of liquid compete against the induced capillary pressure. Hence, the mean imbibition velocity is derived from a balance between the capillary pressure and the viscous losses. The derivation of the mean imbibition velocity is provided in the Supplementary section. Finally, the drying time scale, the time at which the average liquid layer thickness approaches zero (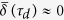), can be estimated by solving Eq. (4) with an initial condition of 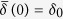
31. If the computed drying time scale (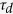) is larger than the surface exposure time to the moving bubble (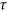
^*^), dryout does not occur.

### Model validation

Since the liquid layer thickness is less than 2 μm and SU8 has a low thermal conductivity, the heating length scale is approximated as the average liquid layer thickness to find a closed form solution for Eq. (4) as follows (further details are provided in the Supplementary section).


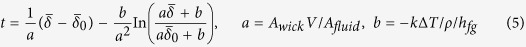


**Figure 6:**
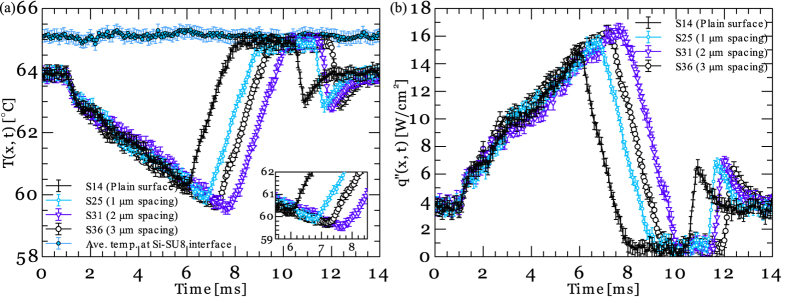
(**a**) Comaprsion of surface temperature-time history and (**b**) local surface heat flux-time history on plain and structured (with pillars spacing 1, 2, and 3 μm) surfaces.

**Figure 7:**
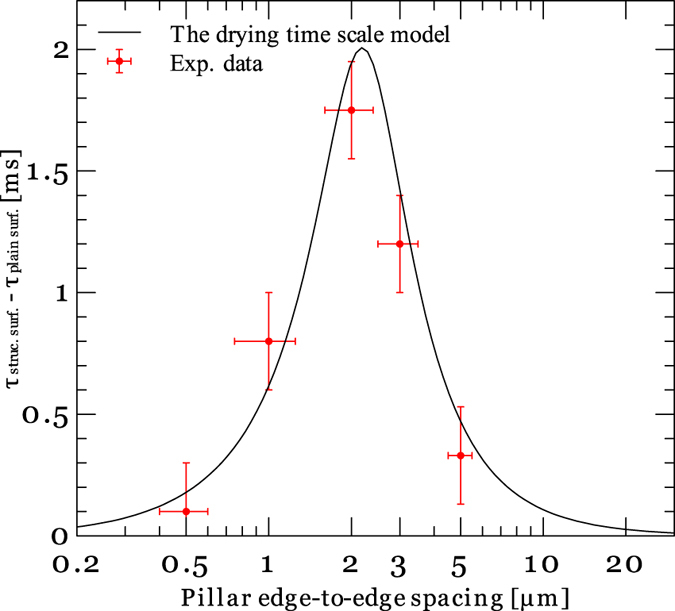
Drying time scale as a function of pillars edge-to-edge spacing.

To evaluate the efficacy of the model presented by Eq. (5), its predictions are compared with the experimental results. Fig. 6a,b show the local temperature and heat flux data measured on textured surfaces with different pillars edge-to-edge spacings. The corresponding difference in the drying time scale of the plain and patterned surfaces are also provided in Fig. 7. As it can be seen, reducing the pillars spacing from 3 μm to 2 μm enhances the thin film evaporation activation time. Conversely, a reduction in the pillar spacing from 2 μm to 1 μm results in a decrease in the drying time scale of the liquid film. Figure 7 also provides the drying time scale results estimated by the model presented in Eq. (5). As it can be seen, the model also predicts a maxima at ~2–3 μm pillars spacing.

This exercise clearly demonstrates the complexity of the surface structures interactions with the fluid dynamics of the microchannel flow boiling process. As the results shown in Figs 3 and 4 illustrate, when the bubble moves along the channel it leaves a relatively thick body of liquid on the corners and sides of the microchannel (note that the channel height is 75 μm). As shown here, only structures with a very narrow pillar spacing range can wick this volume of fluid only over a short distance of ~125 μm because of the poor wickability of FC-72. It is evident that this complex physics could have not been quantified without measurements at the temporal and spatial resolution levels presented in this study; and an optimal pillar spacing of 2 μm would have been very difficult to find through trial and error. It can be argued that in the case of FC-72 and similar liquids, surface structures cannot wick the liquid along the channel length. Hence, enhancements using structures with a substantially different pore size could only be achieved at 1) a particular flow boiling regime in which liquid slugs flow very frequently along the channel at a frequency that matches the liquid film dryout time scale, 2) in an unstable flow where liquid can frequently rewet the channel walls or 3) a regime at which nucleate boiling plays a key role in the overall heat transfer from the surface and the presence of surface structures can promote significant nucleation.

## Implementation of model in design of full-scale heat sinks

Given the stochastic nature of the flow boiling process, one might question the utility of the model in the design of an actual heat sink. To put the developed model to test, a set of experiments were conducted involving microchannel heat sinks with different surface structures and water as the test fluid. The design strategy has been to implement surface structures that can wick water along the channel length. This approach allows access to the main liquid bodies in the manifolds rather a limited amount of liquid left at the channel corners. Prior studies^32^,^33^,^34^,^35^ on wicking characteristics of water within silicon micropillars have indicated that the optimal pillars spacing depends on the wicking length. Hence, the experimental studies discussed in the following section aim at finding the optimal spacing between pillars that can wick water along the channel length from the inlet and exit manifolds.

**Figure 8:**
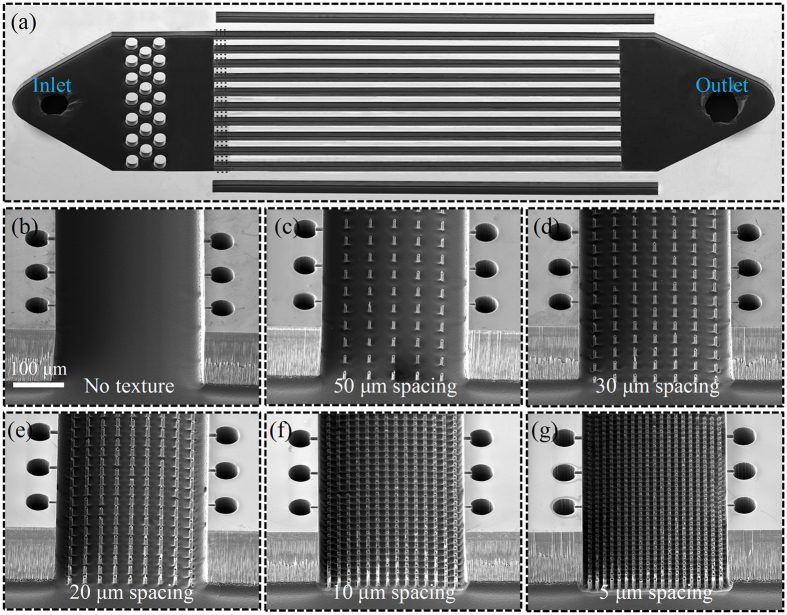
(**a**) A top view SEM of the heat sink and (**b–g**) close up SEM images of the microchannels bottom walls without (**b**) and with (**c–g**) pillars.

The experimental studies are conducted on six microchannel heat sinks. Each heat sink consists of ten parallel microchannels that are 8-mm-long, 300-μm-wide, and 175-μm-tall. The bottom wall of the microchannels is plain in one heat sink and in five others has pillars with a diameter of 10 μm, a height of 20 μm, and an edge-to-edge spacing of 5 to 50 μm. A top view image of the device as well as images of the microchannels bottom walls are shown in Fig. 8. Standard cleanroom microfabrication processes are used to fabricate the heat sinks. A two-step deep reactive ion etching (DRIE) process is used to etch micro-pillars and microchannels in a 500-μm-thick silicon wafer. Re-entrant cavities with three different mouth sizes of 4 μm, 6 μm and 8 μm are integrated in the microchannels sidewalls to promote bubble ebullition process at a wide range of wall superheats. To enhance the flow stability and uniformity, flow restrictors^5^ (three rows of pillars with a diameter of 300 μm and a height of 175 μm) are fabricated at the inlet manifold. In addition, two air gaps are fabricated on two sides of the flow channels to minimize heat spreading through the substrate^36^. The plasma-enhanced chemical vapor deposition (PECVD) process is then utilized to deposit a 300 nm silicon oxide layer on the micro-pillars and the channel sidewalls as a hydrophilic coating and on the backside of the device as an electrical insulation layer. Heat is supplied to the microchannel heat sink area by thin film heaters sputter deposited on the backside of the wafer. To measure the wall temperature, a set of platinum-based thin film resistance temperature detectors (RTDs) is fabricated in between the heaters. The RTDs are calibrated in a constant temperature oven with an accuracy of ± 1.2 °C. Finally, devices are capped by a transparent Polydimethylsiloxane (PDMS) layer.

**Figure 9:**
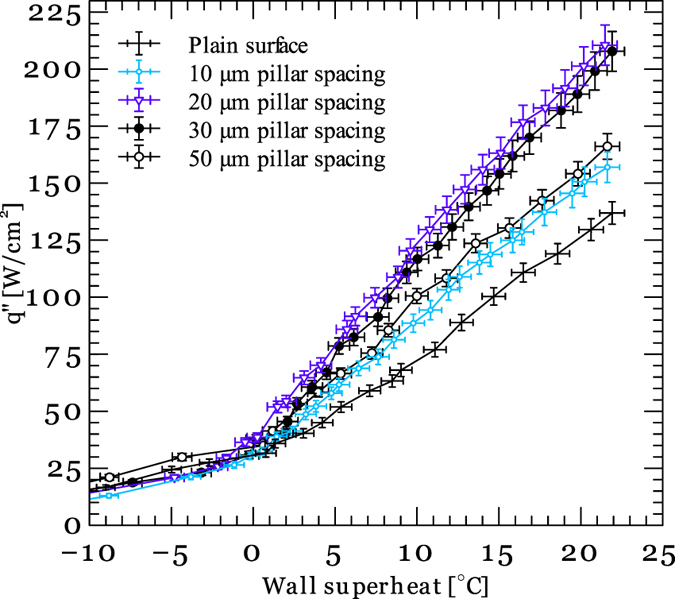
Surface heat flux as a function of wall superheat temperature on plain and textured surfaces at 208 kg/m^2^s.

**Figure 10:**
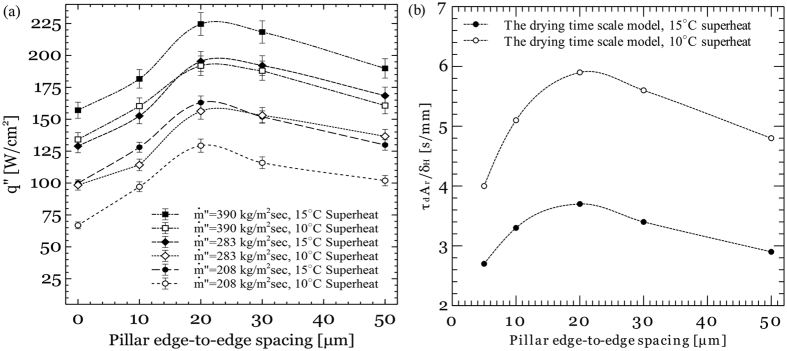
(**a**) Surface heat flux as a function of pillars edge-to-edge spacing at different wall superheats, and (**b**) parameter 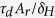 estimated using Eq. (4) as a function of pillars edge-to-edge spacing at different wall superheats.

Tests are conducted in a flow boiling test loop that consists of a liquid reservoir, a water pump and a pre-heater. Tests are conducted at mass fluxes 208, 283 and 390 kg/m^2^s. Figure 9 shows the average surface heat flux as a function of wall superheat temperature at different micropillars spacing and a mass flux of 208 kg/m^2^s. Test results at other mass fluxes are reported in the Supplementary section. The data presented in Fig. 9 are the average obtained from three repetitive experiments. An abrupt change in the curves slope at a few degrees wall superheat indicates transition from single-phase to two-phase boiling regime. It is also evident that heat sinks with micro-textured surfaces deliver a higher heat transfer performance than the plain surface. Figure 10a shows the surface heat flux as a function of pillars spacing at different wall superheats and mass fluxes. It can be seen that decreasing the pillar spacing from 50 μm to 20 μm results in enhancement of the surface heat flux. This is because the capillary pressure generated by the textured surfaces increases as the micro-pillar spacing decreases. However, further reduction in the pillars spacing deteriorates the heat transfer enhancement observed on the textured surfaces. This decline in the average heat flux can be attributed to increase in the viscous pressure losses.

The model presented by Eq. (3) indicates that the heat transfer enhancement observed on a textured surface has a linear relationship with parameter 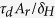. This implies that parameter 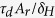 should exhibit the same trend as the average heat flux at different pillars spacing. Figure 10b shows variation of 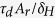 estimated by Eq. (4) as a function of pillars edge-to-edge spacing at two different wall superheats. As it can be seen, 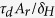 maximizes at a pillar spacing of ~20 μm similar to what is observed in the experimental heat flux data.

## Conclusions

In summary, the physics governing heat transfer enhancement during microscale flow boiling process on textured surfaces is reported. A unique measurement technique was utilized to determine the local heat flux and the drying time scale associated with evaporation of thin liquid films from surface structures fed by the capillary induced micro-inflows. The highly-resolved (spatially and temporally) heat flux data provided by the measurement technique enabled resolving surface dryout time differences as short as 100 μs (note the drying time difference between a surface with 500 nm pillars spacing and a plain surface in Fig. 7). The results showed that due to the poor wickability of FC-72 surface structures can only wick the liquid remained at the microchannel corners over a short distance (~100 μm). This limits the useful pillars spacing to a narrow range between 0.5 to 10 μm with an optima at 2 μm. These findings illustrate the challenges of enhancing the surface heat transfer through incorporation of surface structures in microchannel boiling of low surface tension liquids in the absence of the kind of microscale data and physical insight provided here.

Using the microscale data, a model is proposed that identifies the drying time scale (τ_d_), the heating length scale (δ_H_), and the evaporation area of the thin film (A_r_) as important parameters influencing heat transfer via thin film evaporation process in microchannels flow boiling on textured surfaces. The model proposed for the drying time scale is verified against the drying time scales measured experimentally during combined wicking-thin film evaporation process of FC-72 liquid. The efficacy of the model was examined by conducting a second set of tests on microchannel heat sinks with different surface microstructures and water as the test liquid. The model predicted that enhancement observed on a textured surface has a linear relationship with parameter 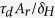. The model suggested that 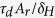 maximizes at a pillar spacing of ~20 μm similar to what was observed in the experimental heat flux data.

The physical insight this study provided on the effects of structures on surface heat transfer enhancement can pave the way for development of next generation high performance two-phase heat sinks.

## Additional Information

**How to cite this article**: Bigham, S. *et al*. Physics of microstructures enhancement of thin film evaporation heat transfer in microchannels flow boiling. *Sci. Rep.* **7**, 44745; doi: 10.1038/srep44745 (2017).

**Publisher's note:** Springer Nature remains neutral with regard to jurisdictional claims in published maps and institutional affiliations.

## Supplementary Material

Supplementary Information
