# Supplementary material for: Physics of microstructures enhancement of thin film evaporation heat transfer in microchannels flow boiling
Source: Sci Rep. 2017 Mar 17;7:44745. doi: 10.1038/srep44745 (PMC5356182; doi:10.1038/srep44745)
Supplement: Supplementary Information [file srep44745-s1.pdf]

SUPPLEMENTARY MATERIALS

Physics of microstructures enhancement of thin film evaporation heat transfer in microchannels flow boiling

Sajjad Bigham, Abdolreza Fazeli and Saeed Moghaddam*

Department of Mechanical and Aerospace Engineering, University of Florida, Gainesville, FL 32611, USA

*Email: saeedmog@ufl.edu

S1. Fabrication of the microfluidic chip with well-defined low-k polymeric nano-pillars

The microfluidic chip was fabricated on a 500-μm-thick silicon wafer through a multistep microfabrication process. A total of 50 resistance temperature detectors (RTDs) consisting of a 50-nm-thick Titanium adhesion layer and a 100-nm-thick Platinum layer were fabricated at Si-SU8 and SU8-fluid interfaces. Nano-pillars made of SU8 polymeric material were fabricated over the top sensor array using electron-beam direct writing. The chip was equipped with a microfabricated pre-heater section made to heat up the working fluid (FC-72) to a desired temperature before entering the test section. The liquid temperature was measured after the pre-heater section by a single RTD sensor positioned between the pre-heater and test section. To control the nucleation site, a 300 nm in diameter cavity was fabricated using a focused ion beam (FIB) milling machine. The cavity was surrounded by a pulsed function microheater fabricated on the SU8 film. To increase the accuracy of the temperature measurements, a 4-wire configuration, also referred to as a Kelvin connection, was utilized. The microfluidic chip was wire bonded to a custom made double sided printed circuit board connected to a high-speed data acquisition system.

Fig. S1 shows the microfabrication sequence. First, 330 nm-thick silicon oxide layers were thermally grown on both sides of an n-type <100> silicon wafer (cf. Fig. S1a). The oxide layers serves as electrical insulation layers between sputtered sensors and bare silicon substrate. Next, the bottom sensor array was fabricated through sputter deposition of 50 nm Ti adhesion layer, 100 nm of Pt layer and 200 nm of Au layer followed by the lift-off process and subsequent Au etching from the serpentine shape part of the sensor array (cf. Fig. S1a). Then, a thin layer of SU8 film was spun coated on the bottom sensor array and subsequently hard baked (cf. Fig. S1b). Next, the top sensor array (50 nm Ti adhesion layer, 100 nm of Pt layer and 200 nm of Au layer) was sputter deposited on the SU8 layer followed by the lift-off process and subsequent Au etching from within the sensor area form the top sensor array (cf. Fig. S1c). It should be noted that the adhesion of a metal layer to a hard baked SU8 is inherently weak. To improve the adhesion strength, the surface of SU8 layer was etched by O2/CF4 reactive ion etching (RIE) with O2 flow rate of 40 sccm, CF4 flow rate of 10 sccm, pressure of 25 mT and power of 100 W to increase its roughness.

In the next step, a blanket Al layer with a thickness of 0.5 μm was sputter deposited on the SU8 film followed by pattering and etching of the Al layer for the bond pads of the bottom sensor array as well as a 10 μm diameter window at the center of the pulsed function micro heater (cf. Fig. S1d). Using the patterned Al layer, SU8 layer was etched by O2/CF4 RIE (cf. Fig. S1e). The Al layer was subsequently etched away from the surface. The inlet and outlet ports were etched from the backside of the Si wafer using the deep reactive ion etching (DRIE) process (cf. Fig. S1f). Then, electron-beam lithography (EBL) technique is employed to pattern the top sensor array with nano-pillars made of SU8 polymeric material (cf. Fig. S1f). Thereafter, a 300 nm in diameter cavity is fabricated using a focused ion beam (FIB) milling machine at the center of the pulsed function micro heater (cf. Fig. S1h). A 75-μm-thick SU8 film was then spun coated to form the microchannel side walls of the microchannel. In the final step, a Polydimethylsiloxane (PDMS) layer was plasma bonded to the SU8 layer to seal the microchannel (cf. Fig. S1i).

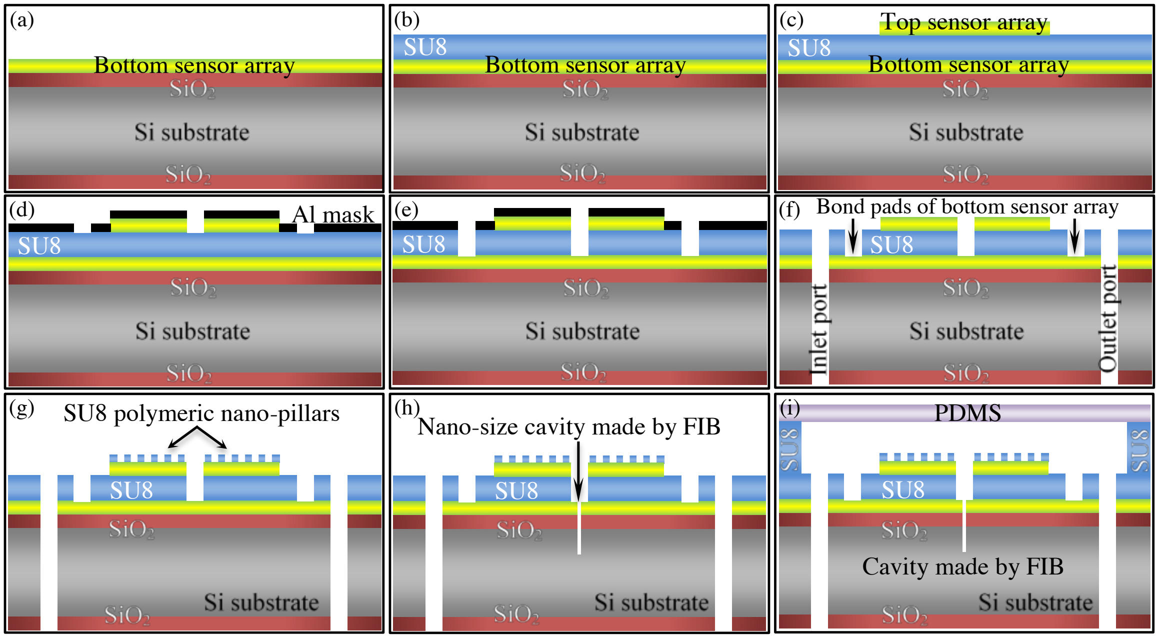

Fig. S1. Fabrication sequence of the microfluidic chip, (a) Thermal SiO2 growth on both sides of Si wafer, deposition of Ti, Pt and Au layers followed by lift-off process and subsequent etching of Au layer from within the bottom sensor array, (b) Spin coat and hard bake the SU8 layer, (c) Sputter deposition of the top sensor array, lift-off process and Au etching from within the senor array, (d) Blanket sputter deposition of Al layer, (e) Pattering and etching of the Al layer, (f) O2/CF4 RIE etching of SU8 layer, (g) DRIE etching of the inlet and outlet ports from the backside of the Si wafer, (h) Fabrication of a nano size cavity using a FIB milling tool, and (i) Spin coating of the microchannel side walls and plasma bonding of a PDMS layer to the SU8 microchannel.

S2. Experimental procedure

Fig. S2 and S3 show a schematic and a photograph of the experimental setup, respectively. The fabricated microchip is wire bonded to a custom made double sided printed circuit board (PCB). Since each RTD sensor has four connections (excitation +/- and channel amplifier +/-), all similar connections from all sensors are guided to a separate 50-pin ribbon socket to be electrically connected to a data acquisition (DAQ) system. The DAQ system, which consists of a current excitation module (NI SCXI-1581), a channel amplifier module (i.e. signal conditioning module) (NI SCXI-1120C), a high speed DAQ module (NI PXI-6289), and a programmable dc power supply module (NI PXI-4110), is commanded by an embedded controller (NI PXI-8115). The temperature data are recorded at a frequency of 20 kHz. The pulsed function micro heater is physically connected to the programmable dc power supply module. All data collection, as well as the control for the applied dc voltage of the pulsed function micro heater, is performed using a LabVIEW program. The thin film heaters are also powered by the NI PXI-4110 dc power supply. A high-speed camera (FASTCAM SA4-Photron) is synchronized with the DAQ to visualize the boiling process at a frequency of 20k frames per second. The working fluid is delivered to the microfluidic chip by a piezoelectric micropump (Model MP6, manufactured by Bartels Mikrotechnik GmbH). Two PX-26 pressure transducers with ±1% reading error are used to measure the pressure drop across the microchannel. The working

fluid is degassed by vigorous boiling for several hours before each experiment. Then, the desired surface temperature is adjusted and allowed about 15 minutes to reach a steady state before recording the data.

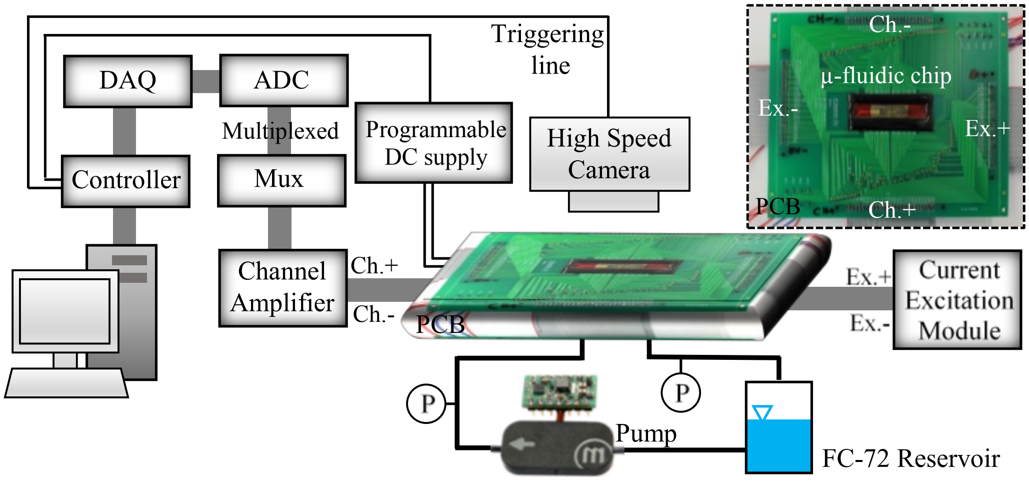

Fig. S2. A schematic of the experimental setup.

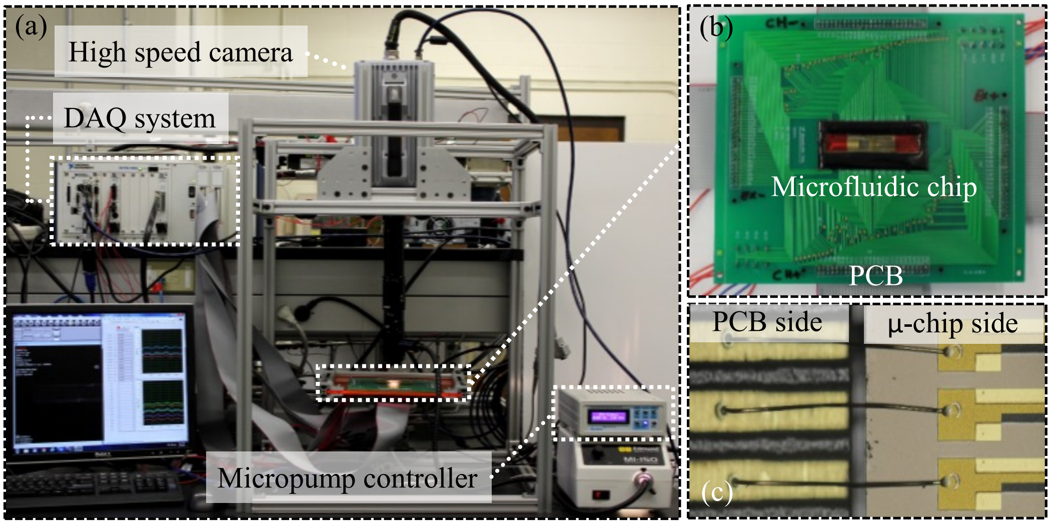

Fig. S3. (a) A photograph of the experimental setup; (b) microfluidic chip wire bonded to a custom made double sided PCB; and (c) a close view of the parallel wire-bonds pattern from PCB to microfluidic chip.

S3. Sensors calibration and uncertainty analysis

The RTD sensors are calibrated prior to the flow boiling experiments to obtain the voltage-temperature relationship of each sensor. The calibration tests are done in a uniform temperature oven for a temperature range of 40 ºC to 90 ºC. A constant current excitation of 100 μA is supplied to each sensor. The temperature sensors have a negligible self-heating. The obtained voltage-temperature curves show a linear trend and the sensitivity of the RTD sensors, the slope of the V-T curves, is 0.13 mV/ºC. The data acquisition system has a maximum uncertainty of ±28 μV, at a gain of 100 with a minimum detectable voltage change of 1 μV. Considering the sensitivity of the sensors and the voltage uncertainty, the maximum error in temperature measurements is determined to be ±0.25 ºC. In addition, the

maximum uncertainty in the measurement of the SU8 film thickness and the local heat flux data are ±0.01 μm and ±1 W/cm^2, respectively.

S4. Mean liquid imbibition velocity into micro- and nano-textured surfaces

Narrow spaces of a textured surface generate capillary pressure due to intermolecular forces between the liquid and the surrounding solid gaps. The capillary pressure induces micro-flows into surface textures, thus replenishing the liquid vaporized during the thin film evaporation process and rewetting of the dry spots. The capillary pressure scales inversely with the texture size. This implies that a surface with narrower spaces has a higher capillary pressure. However, the viscous pressure loss associated with the flow of liquid into surface textures scales inversely with the square of texture size. Therefore, the rate of liquid wicked into surface textures is governed by a balance between the capillary force and the viscous pressure loss. Darcy's law is utilized to relate the mass flow rate through the wick structure to the pressure gradient driving the flow:

$$\dot{m} = -\frac{\rho K A}{\mu}\frac{dP}{dx} \tag{S1}$$

where K is the hydraulic permeability. The net pressure gradient driving the flow can be expressed as:

$$\frac{dP}{dx} = -\frac{P_c}{L_{wick}} \tag{S2}$$

where L_{wick} and P_c are the wicking length and capillary pressure respectively. Combining Eqs. (1) and (2) and expanding $\dot{m}$ as $\rho A V$, we arrive at the following equation:

$$V = P_c K / \mu L_{wick} \tag{S3}$$

where V is the mean imbibition velocity of the liquid wicked into the surface textures. In this study, an analytical model, developed by Dhillon et al. [1], is used to estimate the capillary pressure in the nano-pillar array. In this model, the capillary pressure defined as the change in surface energy per unit volume is written as follows:

$$P_c = \sigma_{lv}\left[4d\cos(\theta_1)/\mathrm{s}(2\mathrm{d}+\mathrm{s}) - (1-\cos(\theta_1)/\mathrm{h}\right],\ \theta_1 = \cos^{-1}(1,(1+4\mathrm{dh}/(\mathrm{s}+\mathrm{h})^2)\cos(\theta)) \tag{S4}$$

where d, s and h are pillar diameter, pillar edge-to-edge spacing and pillar, respectively. The hydraulic permeability of the nano-pillars are also estimated by a model proposed by Byon and Kim [2]. In this model, a surface energy minimization algorithm is utilized to factor in some three-dimensional effects such as finite pillar height and liquid meniscus curvature. The model derived by Byon and Kim [2] is as follows:

$$K = K_{2D}\frac{h_{eff}}{h}\left(\frac{h_{eff}+\frac{\phi d}{4(1-\phi)}}{h+\frac{\phi d}{4(1-\phi)}}\right)\left[1-\frac{\exp\left(2\sqrt{\frac{\phi}{K_{2D}}}h_{eff}\right)-1}{\sqrt{\frac{\phi}{K_{2D}}}h_{eff}\left(\exp\left(2\sqrt{\frac{\phi}{K_{2D}}}h_{eff}\right)+1\right)}\right] \quad \text{(S5)}$$

where

$$h_{eff} = h - d\left\{\begin{array}{l} 0.01476+0.85009\cos\theta+0.215\frac{d}{p}+0.18979\cos^2\theta-3.46929\frac{d}{p}\cos\theta-0.28868\left(\frac{d}{p}\right)^2 \\ +1.05357\frac{d}{p}\cos^2\theta+3.12583\left(\frac{d}{p}\right)^2 cos\theta-1.4243\left(\frac{d}{p}\cos\theta\right)^2 \end{array}\right\}$$

$$\phi = 1-\frac{\pi}{4}\left(\frac{d}{p}\right)$$

S5. Homogenous fluid velocity

To obtain the homogenous fluid velocity, it is assumed that the thin liquid film formed over the surface is constrained within the surface textures. Therefore, the vapor phase travels in the space between the surface textures and the microchannel top wall. The cross-sectional void fraction can be written as follows:

$$\varepsilon = \frac{A_{vapor}}{A_{total}} \quad \text{(S6)}$$

Then, the mean vapor and liquid velocities are given by:

$$U_{vapor} = \frac{\dot{m}_{total}}{\rho_v}\left(\frac{x}{\varepsilon}\right),\ U_{liquid} = \frac{\dot{m}_{total}}{\rho_l}\left(\frac{1-x}{1-\varepsilon}\right) \quad \text{(S7)}$$

where x is the thermodynamic vapor quality. By assuming the vapor and liquid phases travels at the same velocity (i.e., homogenous flow assumption), the vapor quality is obtained as follows:

$$x = \frac{1}{1-\frac{\rho_l}{\rho_v}\left(\frac{1-\varepsilon}{\varepsilon}\right)} \quad \text{(S8)}$$

Therefore, by combining Eqs. (S7) and (S8), the homogenous fluid velocity is derived as follows:

$$U = \dot{m}_{total}\left[\frac{x}{\rho_v}+\frac{1-x}{\rho_l}\right] \quad \text{(S9)}$$

S6. The drying time scale in the case of low-k polymeric nano-pillars

We mentioned that the transient variation in the average liquid layer thickness during combined wicking-thin film evaporation process on a textured surface can be expressed as follows:

$$\frac{d\bar{\delta}}{dt} = \frac{A_{wick}}{A_{proj}} V - \frac{k\Delta T}{\rho h_{fg} \delta_H} \tag{S10}$$

When the surface textures are made of low thermal conductivity materials, the heating length scale equals the average liquid layer thickness. In this case, the above equation is simplified as:

$$\frac{d\bar{\delta}}{dt} = a + \frac{b}{\bar{\delta}},\ a = A_{wick} V / A_{fluid},\ b = -k\Delta T / \rho / h_{fg} \tag{S11}$$

By substituting z for $a\bar{\delta} + b$, we obtain the following equivalent differential equation:

$$\left(\frac{z-b}{z}\right) dz = a^2 dt \tag{S12}$$

By integrating both sides of the above equation and applying an initial boundary equation of $\bar{\delta}(0) = \delta_0$, the drying time scale in the case of low-k polymeric nano-pillars can be expressed as follows:

$$t = \frac{1}{a}\left(\bar{\delta} - \bar{\delta}_0\right) - \frac{b}{a^2} \ln\left(\frac{a\bar{\delta} + b}{a\bar{\delta}_0 + b}\right),\ a = A_{wick} V / A_{fluid},\ b = -k\Delta T / \rho / h_{fg} \tag{S13}$$

S7. Single-phase heat transfer

To estimate the single-phase heat transfer data at different wall superheats, an extrapolation approach is employed. Fig. S4 shows that the equivalent single-phase data associated with wall superheat in the boiling regime are linearly extrapolated from the heat transfer values of the single-phase regime.

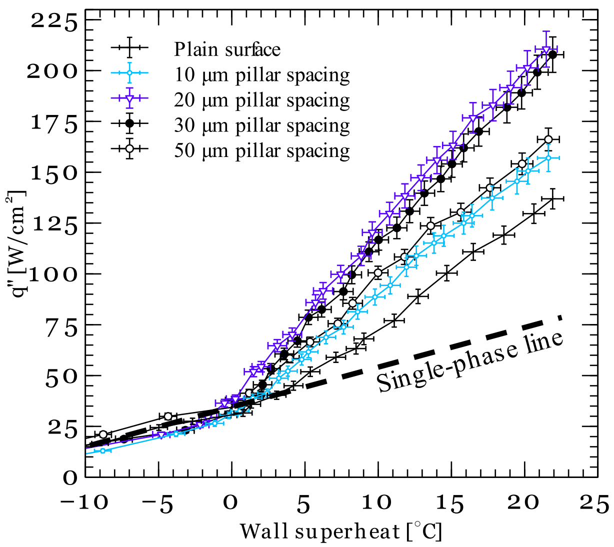

Fig. S4. Average heat flux as a function of average wall superheat on plain and textured surfaces.

References

[1] N.S. Dhillon, J. Buongiorno, K.K. Varanasi, Critical heat flux maxima during boiling crisis on textured surfaces, Nat. Commun. 6 (2015) 8247.

[2] C. Byon, S.J. Kim, The effect of meniscus on the permeability of micro-post arrays, J. Micromechanics Microengineering. 21 (2011) 115011.
